# Pulmonary tuberculosis preventive practices among Anibessa Bus users at Addis Ababa, Ethiopia: a cross-sectional study

**DOI:** 10.1186/s13104-019-4135-1

**Published:** 2019-02-26

**Authors:** Asmamaw Malede, Biruhalem Taye, Mengistu Legesse, Ayal Debie, Agumas Shibabaw

**Affiliations:** 1 College of Medicine and Health Sciences, Wollo University, Dessie, Ethiopia; 2 Akililu Lemma Institute of Pathobiology, Addis Ababa University, Addis Ababa, Ethiopia; 3 College of Medicine and Health Sciences, Addis Ababa University, Addis Ababa, Ethiopia; 4 Department of Health Systems & Policy, College Medicine and Health Sciences, Institute of Public Health, University of Gondar, P.O.Box 196, Gondar, Ethiopia

**Keywords:** PTB, Preventive practices, Factors, Bus users, Ethiopia

## Abstract

**Objective:**

Tuberculosis (TB) is a chronic infectious disease caused by *Mycobacterium tuberculosis*. Smear positive tuberculosis patients are responsible for up to 90% of transmission occurring in the community. However, little is known about pulmonary tuberculosis preventive practices among bus users in Ethiopia. This study aimed to assess the level of Pulmonary Tuberculosis (PTB) preventive practices and associated factors among bus users at Addis Ababa.

**Results:**

Community based cross-sectional study was conducted among bus users at Addis Ababa. Participants were selected using systematic sampling technique. Overall, 50.5% of bus users had good practices on prevention of PTB at Addis Ababa. The odds of practicing prevention of PTB among participants who were attended secondary school (AOR = 4.63; 95% CI 2.62, 11.17) and higher education (AOR = 2.86: 95% CI 1.13, 7.73), resided at Addis Ababa (AOR = 2.51; 95% CI 1.61, 5.21), knowledgeable about PTB (AOR = 4.12; 95% CI 3.14, 5.70), and using mass media (AOR = 2.14; 95% CI 1.78, 4.27) as a source of information were higher than the odds of their respective counterparts. The overall practice of pulmonary tuberculosis prevention among city bus users in the study area was low. Therefore, enhancing educational opportunity and increase community awareness about the causes, risk factors and means of transmission using mass media might improve the practices of PTB prevention during bus transportation.

**Electronic supplementary material:**

The online version of this article (10.1186/s13104-019-4135-1) contains supplementary material, which is available to authorized users.

## Introduction

Tuberculosis (TB) is a chronic infectious disease caused by *Mycobacterium tuberculosis* mainly, and occasionally caused by *Mycobacterium tuberculosis* complex [1]. TB is most commonly transmitted by inhalation of infected droplet nuclei, which are discharged in the air when somebody with untreated sputum-positive pulmonary TB during coughing, sneezing, talking or spiting. In addition, consumption of raw milk containing *Mycobacterium bovis* is also a possible way of getting infected by TB [2]. Smear positive TB patients are responsible for up to 90% of transmission occurring in the community [3].

The national population-based survey in 2011 revealed that the prevalence of smear positive pulmonary TB in Ethiopia among adults and all age groups was 108 and 63 per 100,000 population. The prevalence of smear and/or culture positive confirmed TB for persons aged 15 years and above was 277 per 100,000 [4, 5].

Ethiopia is one of the 27 high burden MDR TB countries ranking 15th with more than 5000 estimated MDR-TB patients annually and one of the four countries in Africa along with South Africa, Nigeria and Democratic Republic of Congo. The estimated MDR TB cases in Ethiopia were 1.6% and 12% among all new and previously treated TB cases, respectively [6]. Ethiopia is one of the high TB, TB/HIV and MDR-TB burden countries worldwide. The estimated TB case detection rate in Ethiopia has been consistently low; the WHO estimates 64% for all forms [6]. The burden of TB is increasing with alarming rate due to various factors including poverty, population growth, migration and HIV/AIDS. But a significant problem lies with the fact that many cases remain undiagnosed [7].

The tubercle bacilli have a high risk particularly in overcrowded settings like areas having no adequate ventilation and light because the bacilli can survive in the dark for long periods and this implies that transmission usually occurs indoors [8]. Casual or random contact, mass public transport and genetic susceptibility are the main factors for epidemics of TB in recent years. Mass public transport is playing the primary role in casually close contact or overcrowded conditions which can facilitate the transmission of PTB [9]. Creating awareness about TB transmission preventive practices especially in high risky or overcrowded conditions were one of the major stop TB strategy to achieve the Millennium Development Goals (MDGs) [10].

However, there was no enough evidence about TB preventive practices particularly among bus users in Ethiopia. Therefore, this study was aimed to address PTB preventive practices and factors associated among Anibessa bus users at Addis Ababa, Ethiopia.

## Main text

### Methods

#### Study setting and design

A community based cross-sectional study was conducted among bus users at Addis Ababa city from March to June, 2012. In the year 2007, the population of the city was estimated to be 2.74 million; from this 52.4% of the residents were females [11].

#### Population and sampling procedure

All Anibessa bus users at Addis Ababa were the source population, while bus users in the selected travel routes of Anibessa bus at Addis Ababa were the study population. Participants who were mentally ill as well as their age less than 18 years during the data collection period were excluded from the study.

Sample size was determined using single population proportion formula with an assumption of proportion (p) = 50%, 95% level of confidence, 5% margin of error (d), and 10% non-response rate. The final sample size was 422. Anibessa city bus service enterprise has a total of 92 travel routes. From these routes, 23 were selected though lottery. Then, the participants were selected using systematic random sampling technique after proportionally allocating the number of participants to each routes of travel based on the bus numbers.

#### Measurements

PTB preventive practices, the dependent variable, was measured by using five item questions and participants who scored above 60% were considered as having good preventive practices, while respondents scored 60% and below of the total practice measuring score were having poor practice. Furthermore, the attitude of participants was measured using twelve item questions and those who scored 60% and above were considered as having favorable attitude towards PTB prevention. On the other hand, the knowledge of respondents about PTB was measured using 20 item questions and those who had scored 60% and above were considered as knowledgeable.

#### Data collection tools and quality control

Data was collected through semi-structured interviewer administered questionnaire adopted from reviewing different literatures. The questionnaire was first prepared in English language, and translated to Amharic and finally, back to English in order to ensure its consistency. Pretest was conducted among 20 participants and necessary modification was made based on the pretest findings. Six clinical nurses for data collectors, and 2 health officers for supervisors were recruited. A day training was given to data collectors and supervisors on the basic techniques of data collection. The data was cleaned by supervisors and principal investigators on daily basis for ensuring its completeness and consistency.

#### Data management and analysis

The data was entered and analyzed using Epi info version 7 and STATA Version 13, respectively. Descriptive statistics such as mean, frequency, and percentages were presented using tables, and figures. Binary logistic regression model was used to identify factors significantly associated with PTB preventive practices. Adjusted odds ratio (AOR) with 95% confidence interval (CI) and p-value < 0.05 were used to determine the strengths and factors associated with PTB preventive practices.

### Results

#### Socio-demographic and economic characteristics

A total of 408 Addis Ababa city bus users were participated with a response rate of 96.7%. The median age of the participants were 25 years with interquartile range of 12.25. Nearly, two-thirds of the participants were males and unmarried. About 44.6% were resided in Addis Ababa and 70.1% of bus users were Orthodox Christian. More than one-third of respondents reported that they always had used Anibessa bus. More than half of the participants monthly household income were less than ETB 500 (Table 1).

**Table 1:** Socio-demographic and economic characteristics of bus users at Addis Ababa, 2012

Variables	Category	Frequency	Percent (%)
Sex	Male	279	68.4
	Female	129	31.6
Age group (in years)	18–25	200	49.0
	26–35	127	31.1
	≥ 36	81	19.9
Religion	Orthodox	286	70.1
	Catholic	6	1.47
	Muslim	60	14.7
	Protestant	56	13.7
Marital status	Single	247	60.5
	Married	161	39.5
Ethnicity	Amhara	171	41.9
	Oromo	122	29.9
	Gurage	75	18.4
	Tigray	16	3.9
	Others	24	5.9
Educational status	Unable to read and write	59	14.5
	Able to read and write	35	8.6
	Primary school	81	19.9
	Secondary school	88	21.6
	College and above	145	35.5
House hold monthly income	ETB < 500	211	51.7
	ETB 500–1000	98	24.0
	ETB ≥ 1000	99	24.3
Occupation	Student	57	14.0
	Merchant	35	8.6
	Unemployed	127	31.1
	Government employed	71	17.4
	Private employed	94	23.0
	Others	24	5.9
Residence	Out of Addis Ababa	226	55.4
	Addis Ababa	182	44.6
Frequency of bus use	Some times	251	61.5
	Always	157	38.5

#### Knowledge of participants about PTB

Nearly, two-third participants reported that cough of 2 weeks and bloody sputum were among the major symptoms of pulmonary tuberculosis, respectively. Moreover, 52.8, 55.65, and 52.7% of bus users stated that closing of bus windows during travelling, coughing and sneezing could transmit PTB, respectively. On top of this, more than half of participants recognized that PTB could be caused by germs or bacteria’s, and about 62.3% described overcrowding condition such as using city bus might be a risk factor for PTB transmission and 96.8% of participants described that PTB is a curable disease. More than half (52.7%) of the respondents were knowledgeable about the causes, risk factors, means of transmission, signs, and symptoms of pulmonary tuberculosis (Table 2).

**Table 2:** Knowledge of participants about PTB among bus users at Addis Ababa, 2012

Variables	Frequency			
	Yes	%	No	%
*Signs and symptoms of PTB*				
Cough of 2 weeks	261	64.0	147	36.0
Bloody sputum	247	60.5	161	39.5
Weight loss	201	49.3	207	50.7
Loss of appetite	204	50.0	204	50.0
*Mode of PTB transmission*				
Closing of bus windows	240	58.8	168	41.2
Inadequate ventilation and light	43	10.5	365	89.5
Coughing	227	55.6	181	44.4
Talking	197	48.3	211	51.7
Sneezing	215	52.7	193	47.3
Lip kissing	175	42.9	233	57.1
Improper disposal of sputum	243	59.6	165	40.4
*Causes and risk factors for PTB*				
Germs/bacteria is a cause for TB	227	55.6	181	44.4
Cigarette smoking	122	29.9	286	70.1
Overcrowding situation	254	62.3	154	37.8
PLHIV	249	61.0	159	39.0
Poor people	177	43.4	231	56.6
Prisoners	155	38.0	253	62.0
*Rx of PTB*				
TB is curable	395	96.8	13	3.2
Defaulting anti-TB causes incurable	274	67.2	134	32.8
Defaulting anti-TB causes drug resistance	190	46.6	218	53.4
Knowledgeable about PTB	215	52.7	193	47.3

#### Source of information and attitude towards prevention of TB

More than 95% of the participants were heard about the disease pulmonary tuberculosis. The source of information for more than 80% of bus users were mass media. Furthermore, about 64% of the participants who had unfavorable attitude towards PTB preventive practices (Additional file 1: Figure S1).

#### PTB preventive practices

About 55% of bus users were covering their mouth and nose during coughing and sneezing for preventing PTB transmission during transportation. More than 19% of participants were opening windows and about 50% of bus users were properly disposing their sputum, tried to get early diagnosis and treatment when suspecting the disease. Nearly, half of the participants (50.5%) had good practices on prevention of PTB among bus users in the study area (Additional file 2: Figure S2).

#### Factors associated with PTB preventive practices

Binary logistic regression model was used for multivariable analysis. Furthermore, AOR and p-value less than 0.05 were used to determine the strength and factors associated with PTB preventive practices. The odds of practicing prevention of PTB among participants attended secondary school (AOR = 4.63; 95% CI 2.62, 11.17) and higher education (AOR = 2.86; 95% CI 1.13, 7.73) were 4.63 and 2.86 times higher than the odds of participants who were unable to read and write, respectively. The odds of practicing prevention of PTB among respondents resided at Addis Ababa (AOR = 2.51; 95% CI 1.61, 5.21) were 2.51 times higher than the odds of practicing among participants resided out of Addis Ababa. The odds of practicing prevention of PTB among knowledgeable bus users about PTB (AOR = 4.12; 95% CI 3.14, 5.70) and participants using mass media (AOR = 2.14; 95% CI 1.78, 4.27) as a source of information were 4.12 and 2.14 times higher than that of the odds of practicing with their respective counterparts (Table 3).

**Table 3:** Factors associated with PTB preventive practices among bus users at Addis Ababa, 2012

Variables	Category	PTB preventive practice		COR (95% CI)	AOR (95% CI)
		Good	Poor		
Educational status	Unable to read and write	17	42	1	1
	Able to read and write	3	32	0.23 (0.06, 0.86)	0.21 (0.08, 1.31)
	Primary school	30	51	1.45 (0.71, 2.99)	1.37 (0.59, 3.87)
	Secondary school	64	24	6.59 (3.16, 13.71)	4.63 (2.62, 11.17)*
	College and above	92	53	4.29 (2.22, 8.27)	2.86 (1.13, 7.73)*
Residence	Out of AA	97	129	1	1
	AA	109	73	1.99 (1.34, 2.95)	2.51 (1.61, 5.21)*
Sex	Male	140	139	1	1
	Female	66	63	1.04 (0.69,1.58)	1.18 (0.87,2.68)
Age in years	18–25	94	106	1	1
	26–35	67	60	1.26 (0.81,1.97)	1.15 (0.69,1.68)
	≥ 36	45	36	1.41 (0.84,2.37)	1.04 (0.57,1.91)
Marital status	Unmarried	119	128	1	1
	Married	87	74	1.26 (0.85,1.88)	1.01 (0.61,1.65)
Knowledge about PTB	Not knowledgeable	72	121	1	1
	Knowledgeable	134	81	2.78 (2.12,3.84)	4.12 (3.14,5.70)*
Attitude towards PTB prevention	Unfavorable	127	135	1	1
	Favorable	79	67	1.25 (0.83,1.88)	1.36 (0.89,2.10)
Using mass media	No	28	42	1	1
	Yes	178	160	1.67 (1.17, 3.19)	2.14 (1.78, 4.27)*

* Significant at p-value < 0.05

### Discussion

This study aimed to assess the level of PTB preventive practices and associated factors among Anibessa bus users at Addis Ababa, Ethiopia. The finding showed that nearly half of the participants (50.5%) had good PTB preventive practices. This was comparable with the study done in Thailand refugees (55.5%) [12]. Furthermore, this result was lower than that of studies conducted in Eastern Amhara region covering mouth and nose practice while coughing (66.6%) [13] and Tarlac city (61%) [14]. However, it was higher than studies conducted in Eastern Amhara region (45.3%) [13], Gambella (45.5%) [15], Iraq (38.2%) [16], and Nigeria (32%) [17]. The possible explanation for this difference might be due to difference in study design, area, period as well as participants.

The odds of practicing prevention of PTB among participants attended secondary school and higher education were 4.63 and 2.86 times higher than the odds of participants who were unable to read and write, respectively. This find was consistent with studies done in Tigray, Ethiopia, Kenia, and India [18–20]. The possible explanation might be due to respondents attended secondary and above education could be analyzed the economic burden and psychological consequences of the disease. The other justification could be educated bus users might have positive attitude to apply preventive practices of PTB.

The odds of practicing prevention of PTB among respondents resided at Addis Ababa were 2.51 times higher than the odds of practicing among participants resided out of Addis Ababa. This might be due to accessibility of getting information about pulmonary tuberculosis can be relatively high as a result of the availability of health facilities and mass media in Addis. The other justification might also participants in Addis could be educated and might know the means of prevention for PTB transmission.

Despite a higher proportion of the participants have heard about TB, but nearly half of the respondents had poor knowledge about the cause, risk factors, mode of transmissions, treatments, symptoms, and signs of the disease. Moreover, this study revealed that the study participants had basic knowledge about the symptoms of TB and its modes of transmission. This result was consistent with the studies in Crotia, Madagascar, Ethiopia, Brazil, and India [21–25]. The most frequently reported symptom by the participants was coughing. The odds of practicing prevention of PTB among knowledgeable bus users about PTB were 4.12 higher than that of the odds of practicing with their counterparts. This finding was supported by studies conducted in Gambella, Ethiopia, Tajikistan, and Nigeria [15, 17, 26]. The possible explanation might be due to bus users who know about the causes, risk factors, means of transmission, and prevention might prone to practice their knowledge into practice.

Most of bus users mentioned that mass media was their main sources of information about TB which was similar to other findings in Lahore, Iraq, and Pakistan [14, 16, 27]. The odds of practicing prevention of PTB among participants using mass media as a source of information were 2.14 times higher than that of the odds of practicing with non-users. This might be due to participants might give a chance to understand well on how to practice prevention of PTB and analyze the social, economic and health related crises as a result of acquiring the disease.

The overall practice of pulmonary tuberculosis prevention among city bus users was low. Therefore, educating the community to cover their mouth and nose during coughing and sneezing. Accordingly, awareness creation of bus users about proper disposal of their sputum and to open the bus windows during transportation. Additionally, community awareness about the causes, risk factors and means of transmission might improve the practices of PTB prevention during bus transportation.

## Limitation

- The cross-sectional study could not assess temporality of the cause-effect relationships.
- The 95% confidence intervals for the odds ratios for some variables was too wide, this might lower the power of association.

## Additional files

**Additional file 1: Figure S1.** Source of information and attitude towards PTB preventive practices among bus users at Addis Ababa, 2012.

**Additional file 2: Figure S2.** PTB preventive practices among bus users at Addis Ababa, Ethiopia, 2012.

## Abbreviations

AA: Addis Ababa
AOR: adjusted odds ratio
CSA: Central Statistical Agency
COR: crude odds ratio
MDGs: Millennium Development Goals
PTB: pulmonary tuberculosis
Rx: treatment
TB: tuberculosis
WHO: World Health Organization
