# Supplementary figures and images for: Pulmonary tuberculosis preventive practices among Anibessa Bus users at Addis Ababa, Ethiopia: a cross-sectional study
Source: BMC Res Notes. 2019 Feb 26;12:104. doi: 10.1186/s13104-019-4135-1 (PMC6390587; doi:10.1186/s13104-019-4135-1)

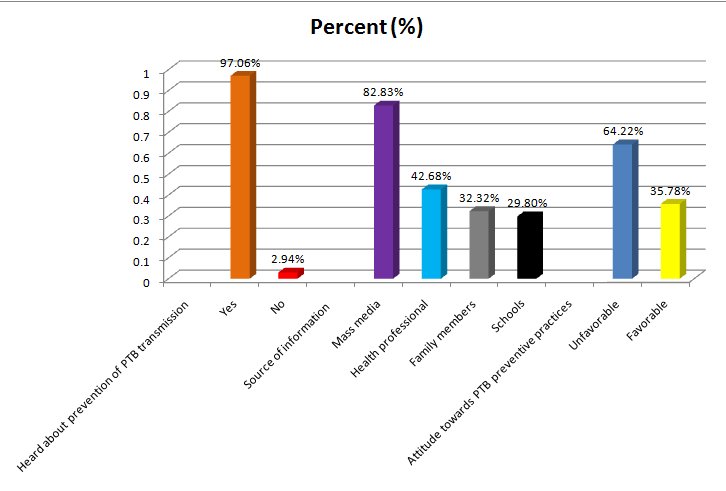

Supplement: Supplementary file 1 — Additional file 1: Figure S1. Source of information and attitude towards PTB preventive practices among bus users at Addis Ababa, 2012. [file 13104_2019_4135_MOESM1_ESM.tif]

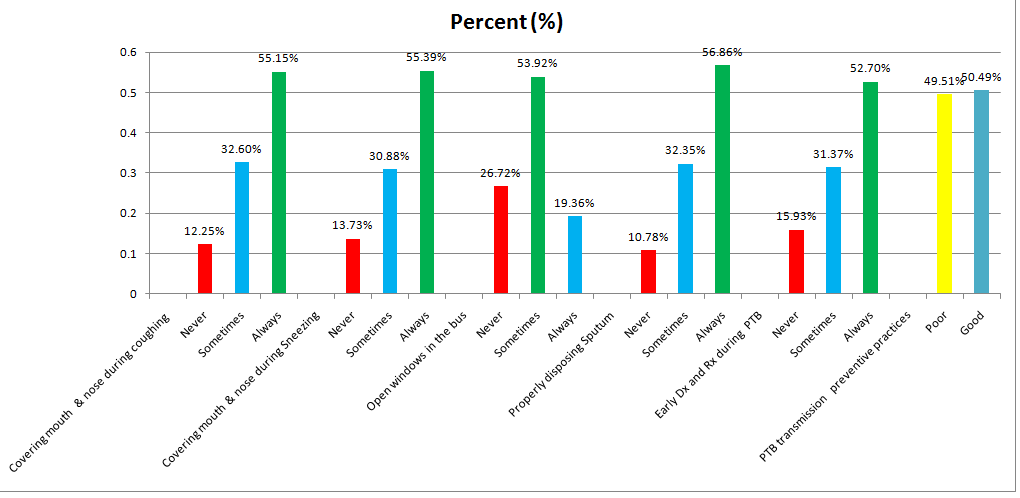

Supplement: Supplementary file 2 — Additional file 2: Figure S2. PTB preventive practices among bus users at Addis Ababa, Ethiopia, 2012. [file 13104_2019_4135_MOESM2_ESM.tif]
